# A case report of nocardiosis at the cauda equina

**DOI:** 10.1007/s00776-013-0460-8

**Published:** 2013-09-10

**Authors:** Kurimoto Hisatsugu, Hirabayashi Shigeru, Miura Makoto, Yamamoto Iwao, Yamada Kazuaki, Matsushita Takashi

**Affiliations:** Department of Orthopaedic Surgery, Teikyo University Hospital, 11-1 Kaga-2 Chome, Itabashi, Tokyo, Japan

## Introduction

Nocardiosis is an infectious disease caused by an aerobic Gram-positive bacillus belonging to the Actinomycetales family that is broadly distributed in soil. Like other bacilli, *Nocardia* species cause disease mainly in immunocompromised patients, such as renal transplant recipients and those with diabetes mellitus [1–3]. Skin and lung are the usual sites of primary infection [4]. The lungs are the most common site of primary infection, and, in such cases, patients usually complain of fever and cough. Gradually, the bacillus spreads through the hematogeneous route to all regions of the body. Approximately one-half of patients with systemic nocardiosis have central nervous system involvement. *Nocardia* can invade the brain asymptomatically and lodge there silently for months or years. However, spinal cord involvement is very rare [5].

There are few reports concerning involvement of the spinal cord, and, to the best of our knowledge, there is only one previous report of abscess of the cauda equina caused by *Nocardia* in an immunocompetent host [1].

In this report, we present a very rare case of *Nocardia* infection of the cauda equina, which was initially diagnosed as a tumor because of a lack of infectious systemic symptoms and relevant past history; multiple abscesses in the brain and the lung portal area were revealed after surgery was performed.

## Case report

The patient was a 69-year-old female who presented with chief complaints of incomplete paralysis of the right lower extremity and difficulty walking. She had a history of anxiety, and had been administered tranquilizers. There was no other pertinent past medical history. She had no history of employment and the family medical history was negative.

## History of the present illness

In April 2012, the patient experienced lower back pain and was diagnosed with lumbar spondylolysis at another hospital. Conservative outpatient treatment did not improve the pain, and after 7 days incomplete paralysis of the right lower extremity was noted. On magnetic resonance imaging (MRI), a mass in the cauda equina area was revealed and was initially diagnosed as an intradural, extramedullary tumor. On the following day, the patient experienced difficulty walking and was admitted to our hospital.

## Neurological and laboratory findings on admission

There was spontaneous pain in the lower lumbar region independent of mobility. The patient was afebrile, and there was no tenderness over the spinous processes or paravertebral muscles.

Muscle weakness was severe in the right lower extremity; muscle power by manual muscle testing (MMT) was grade 0–1 for the tibialis anterior (TA), grade 2 for the extensor hallucis longus (EHL) and the extensor digitorum longus (EDL), grade 2 for the peroneus, grade 0–1 for the gastrocnemius, and grade 3 for the hamstrings. No muscle weakness in the left lower extremity was found. There was a sensory disturbance in the right fifth lumbar (L5) root area. Deep tendon reflexes were absent in both lower extremities. No bladder or bowel disturbances or neurological abnormalities of the cranial nerve root areas were found.

Laboratory data was as follows:

- Hemoglobin (Hb) 12.5 g/dl, white blood cell (WBC) 5,000/μl
- Platelet (PLT) 354,000/μl, blood urea nitrogen (BUN) 11.0 mg/dl
- Creatinin (Crea) 0.7 mg/dl, sodium (Na)137 mEq/l, potassium(K) 4.0 mEq/l

There were no remarkable laboratory findings except a slight elevation of C-reactive protein (CRP), to 0.77 mg/l.

## Imaging findings on admission

**Fig. 1:**
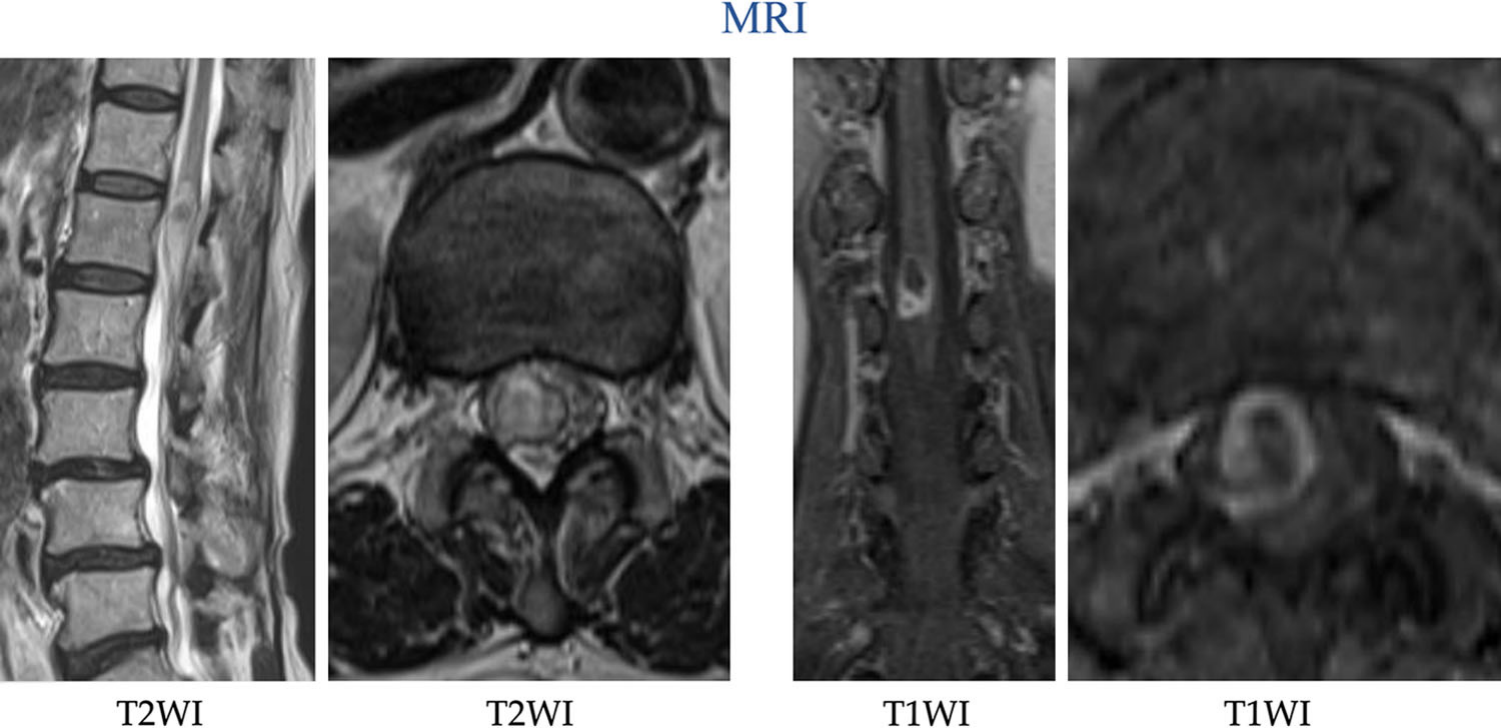
*Left* T2-weighted MRI. Slight spondylolysis at both L3 and L4 was found; however, there was no lumbar spinal canal stenosis. At the T12/L1/L2 level, a spindle-shaped intradural lesion was revealed on a sagittal T2-weighted image. The entire outer area of the lesion was high-intensity, and a round inner area was low-intensity. On an axial T2-weighted image, the lesion was seen at the right side within the dura mater, causing the nerve filaments to shift to the left. The margin of the lesion was irregular and of low intensity, while the inner area was mixed high- and iso-intensity. *Right* enhanced T1-weighted MRI. Only the margin of the lesion was enhanced

On MRI, slight spondylolysis at both L3 and L4 was found, and there was no lumbar spinal canal stenosis. At the T12/L1/L2 level, a spindle-shaped intradural lesion was seen on a sagittal T2-weighted image. The entire outer area of the lesion was high-intensity, and a round inner area was low-intensity. On an axial T2-weighted image, the lesion was seen at the right side within the dura mater, causing the nerve filaments to shift to the left. The margin of the lesion was irregular and of low intensity, while the inner area was mixed high- and iso-intensity (Fig. 1, left). On an enhanced T1-weighted image, only the margin of the lesion was enhanced (Fig. 1, right).

The lesion was suspected to be an ependymoma, an astrocytoma, or a hemorrhage within the cauda equina.

## Presurgical clinical course

Seventeen hours after admission, the area of muscle weakness progressed upward to the level of the iliopsoas (IP) and the area of sensory disturbance also gradually spread. The MMT muscle power of the IP and the quadratus femoris (Q) decreased from normal to grade 3. Despite administration of high-dose methylprednisolone, the paraplegia worsened, and difficulty with urination occurred during the first 30 h after admission. Accordingly, an emergency operation was performed.

## Surgery

**Fig. 2:**
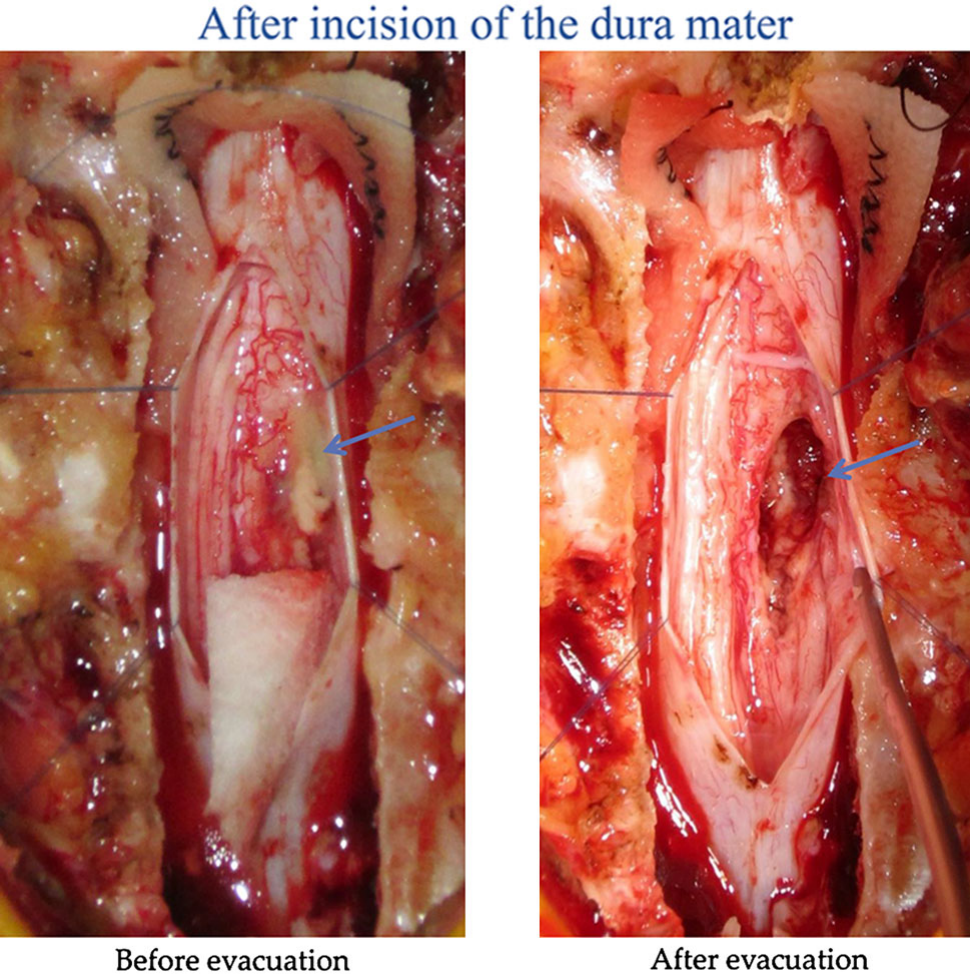
Intraoperative photographs. *Left* after incision of the lesion, there was an outflow of pus. *Right* debris covering the inner surface of the mass was evacuated to the greatest extent possible

Laminectomy was performed at the T12/L1/L2 level. A part of the surface of the cauda equina was revealed to be yellow in color and expanded. After incision of the lesion, there was a sudden and unexpected outflow of pus (Fig. 2, left). The pus was located within the cauda equina, and debris resembling necrotic tissue covered the inner surface of the mass; the abscess was evacuated to the greatest extent possible (Fig. 2, right).

**Fig. 3:**
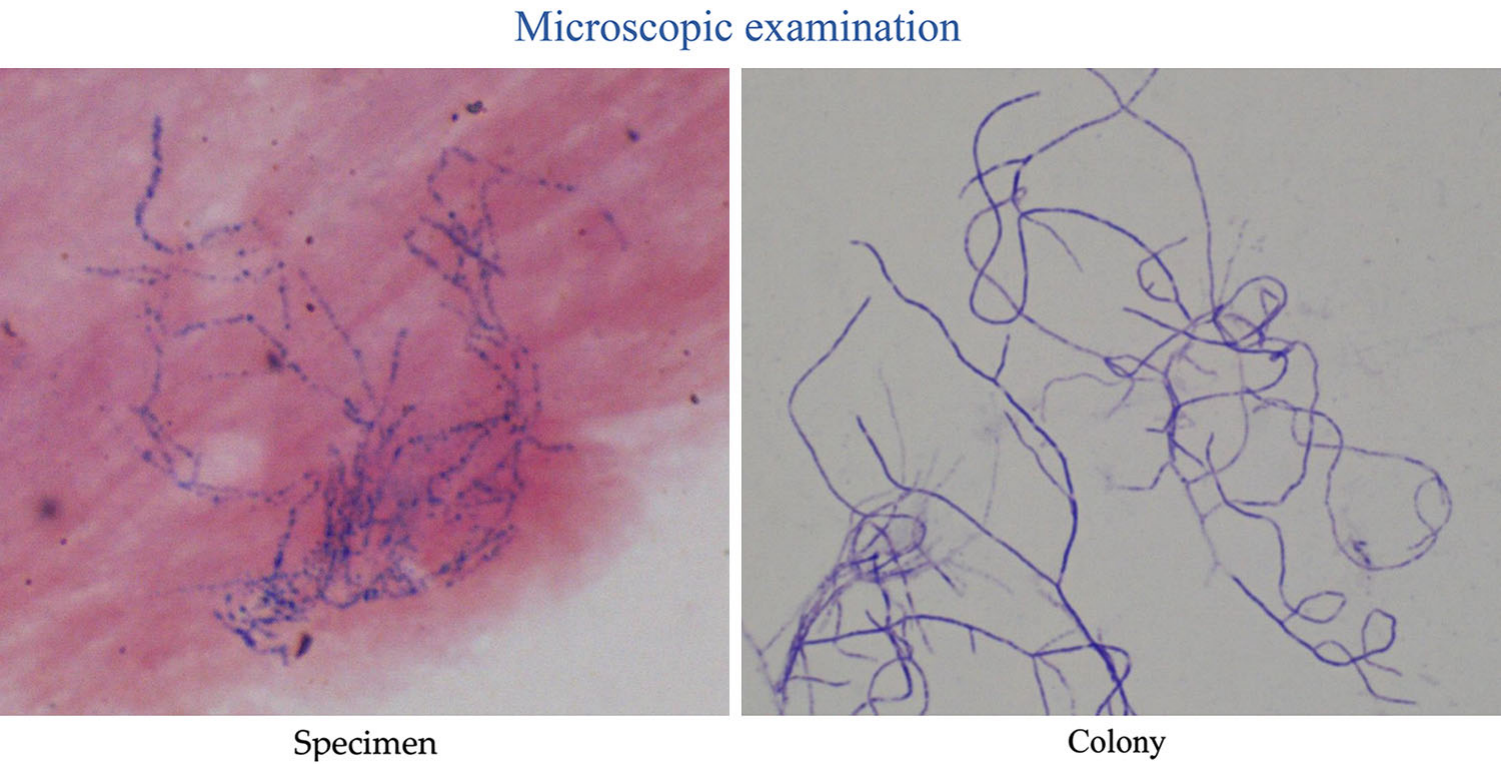
Microscopic examination (Gram stain). Multiple colonies were found. Irregularly weakly- and strongly-stained, beaded, branching, thin filaments were observed

A bacterial examination of the debris revealed colonies of a Gram-positive bacillus, and on microscopic examination, irregularly weakly- and strongly-stained, beaded, branching, thin filaments were observed (Fig. 3). *Nocardia beijingensis* was confirmed by culture.

## Postoperative clinical course

**Fig. 4:**
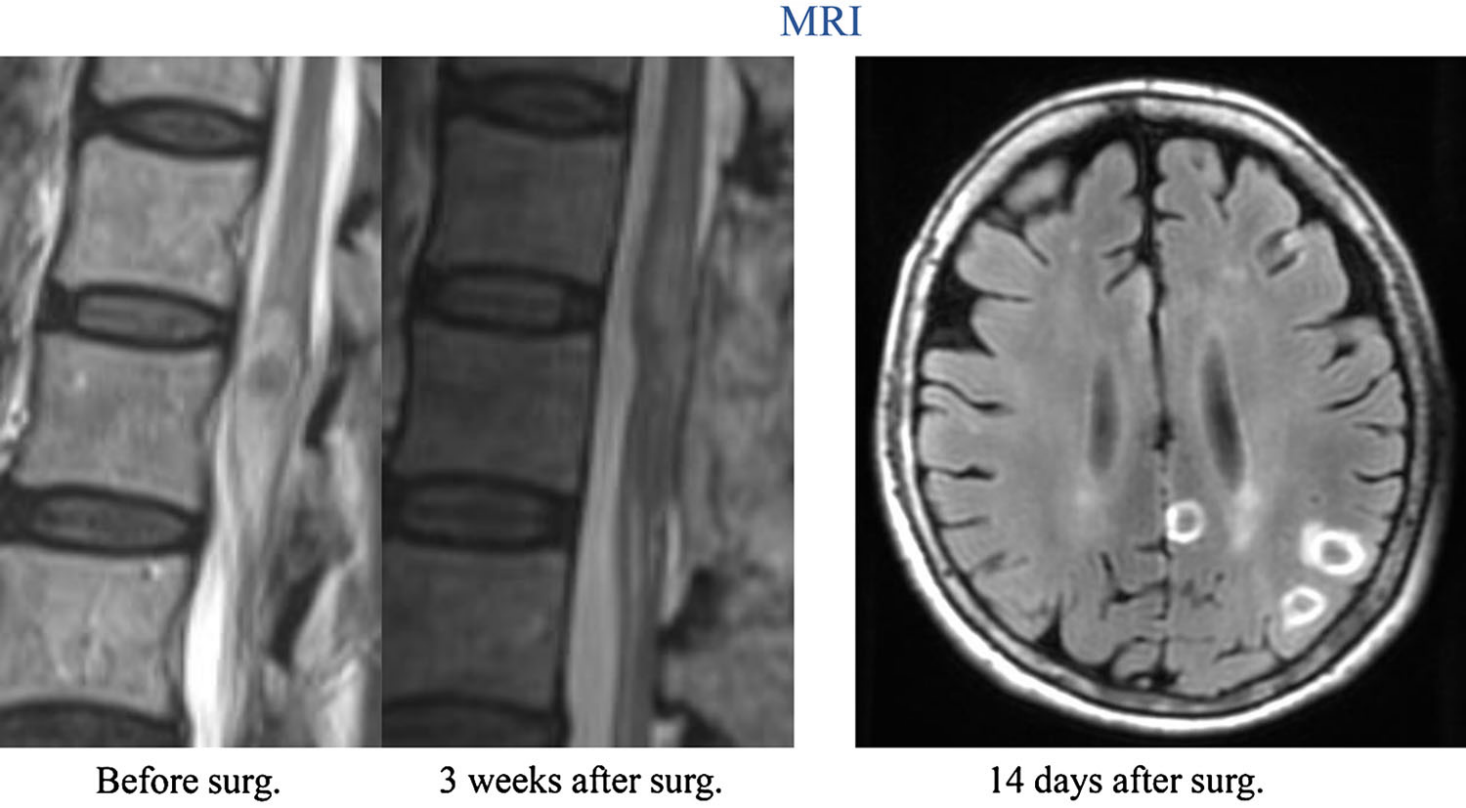
Postoperative MRI. *Left* after surgery, the abscess in the cauda equina was confirmed to have been completely resected. *Right* multiple brain abscesses were identified

**Fig. 5:**
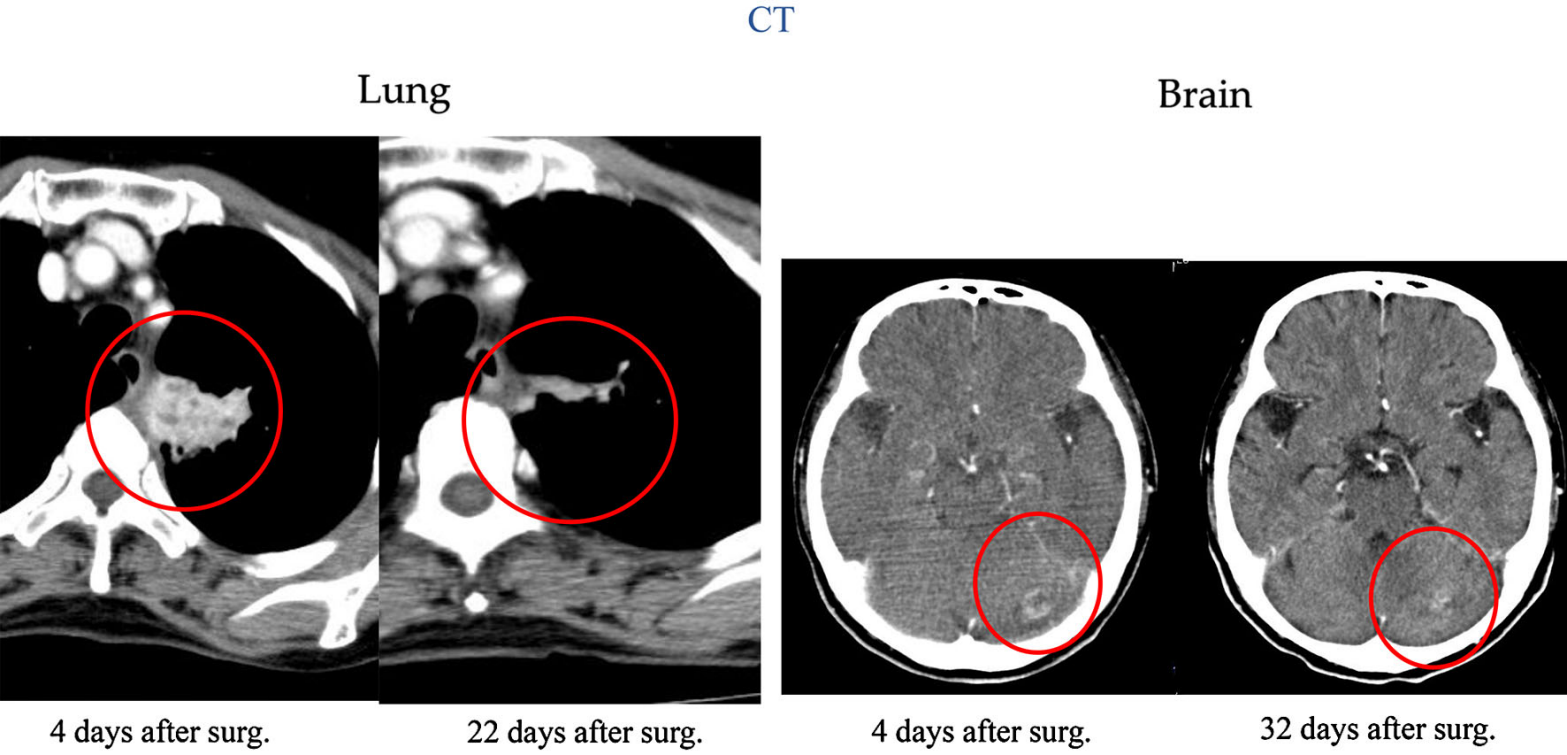
Follow-up CT. *Left* on postoperative day 4, an abscess was found at the left lung portal area, and by day 22, the abscess had decreased in size. *Right* a brain abscess, which was identified 1 day after surgery. By postoperative day 32, the abscess had decreased in size

An MRI on postoperative day 21 revealed that the cauda equina abscess had been completely resected (Fig. 4, left). As *Nocardia* infection had been confirmed, a whole-body computed tomography (CT) scan was performed on postoperative day 4, which revealed multiple lesions, thought to be abscesses, within the brain and the left lung portal area (Fig. 5, left); a brain MRI performed on postoperative day 14 confirmed presence of abscesses (Fig. 4, right).

From postoperative days 5–14, composite sulfamethoxazole-trimethoprim (ST) was administered via intravenous injection to provide a trimethoprim (TMP) component of 600 mg per day, and then from days 14 to 56 the comparable dose was administered orally. After day 56, the dose was reduced to TMP component of 400 mg per day, and minomycin (MINO) 100 mg per day was added. On postoperative day 14, laboratory data showed that the CRP had normalized (0.02 mg/l), and CT on postoperative days 22 and 32 revealed that the brain and lung lesions had decreased in size (Fig. 5).

On postoperative day 40, the muscle power of the IP and the Q had improved to grade 4 by MMT; however, the muscles below the TA remained weak, at grade 0–1, and the sensory disturbance and bladder/bowel disturbance were unchanged. Currently, 7 months after surgery, the sensory disturbance and bladder/bowel disturbance are improving, the TA power has improved to grade 4, and the patient is ambulatory using crutches while undergoing rehabilitation for walking.

## Discussion

### *Nocardia* infection

Nocardiosis is caused by *Nocardia* species, which comprise at least three and perhaps six major subgroups, including *Nocardia asteroids* sensu stricto, *Nocardia farcinica*, and *Nocardia nova* [5]. Lung and skin are the most commonly affected primary sites [4]. In two-thirds of patients, the lung is the primary site of infection [6], and bacilli are distributed systemically by the hematogeneous route. Sixty-four percent of patients are immunocompromised hosts [7]. By microscopic examination, the findings of irregularly weakly- and strongly-stained, beaded, branching, thin filaments are extremely important for identification of *Nocardia* species [5].

According to Beaman et al. [7], central nervous system involvement of *Nocardia* can be seen in almost 50 % of patients with disseminated nocardiosis, and an intracerebral parenchymal abscess can form in any region of the brain. Lerner [5] also reported that approximately 45 % of patients with systemic nocardiosis have central nervous system infections. However, spinal cord involvement is very rare.

### Spinal cord involvement of *Nocardia*

There are few reports concerning abscess of the spinal cord caused by this bacillus, and, to the best of our knowledge, there is only one previous report of *Nocardia* in the cauda equina. Lee et al. [1] reported an 82-year-old man with diabetes mellitus who suffered a solitary abscess of the conus medullaris and underwent surgery.

The salient features of the current case were as follows: (1) the brain and lungs were infected, but the patient had no symptoms relating to these lesions, (2) the patient was not immunocompromised, and (3) there were no abnormalities of laboratory data except a slight elevation of CRP. Therefore, initially the lesion was diagnosed as a spinal tumor. Subsequently, abscesses were found at not only in the cauda equina but also in the brain and the portal area of the lung. Probably, the lung was the primary site of infection, as in most cases, and it disseminated to the brain hematogeneously. From there the bacillus might have descended to the cauda equina via the central canal of the spinal cord. This hypothesis is substantiated by the fact that the pus was located within the cauda equina.

### Treatment of *Nocardia* infection

For an abscess in the spinal cord, surgical decompression and drainage followed by intravenous administration of effective antibiotics is the standard treatment. For infection by *Nocardia*, the first-choice antibiotic is usually composite ST, but the selection of antibiotics should be determined according to the sensitivity and tolerance of the isolated organism. Sometimes, amikacin, imipenem, minocycline, or a combination of amoxicillin and clavulanic acid have been administered. Recently, the efficacy of linezolid has been reported [9].

The administration of antibiotics must usually be continued for at least 6 months. However, in patients with an infected central nervous system, those with multiple infections and patients with immuno-insufficiency, the administration must be continued for at least 1 year [4, 5, 8].

## Conclusion

In this report, we present a very rare case of nocardiosis that manifested initially as a tumor-like configuration in the cauda equina. The differential diagnosis for an intradural lesion at the level of the cauda equina must include abscess, even when obvious signs and symptoms of infection are not evident.

The patient was informed that we wanted to submit data from her case for publication, and provided her consent.
